# Mesenchymal stromal cells inhibit CD25 expression via the mTOR pathway to potentiate T-cell suppression

**DOI:** 10.1038/cddis.2017.45

**Published:** 2017-02-23

**Authors:** Hyun Seung Yoo, Kyuheon Lee, Kwangmin Na, Yong Xu Zhang, Hyun-Ja Lim, TacGhee Yi, Sun U Song, Myung-Shin Jeon

**Affiliations:** 1 Department of Molecular Biomedicine, Translational Research Center, Inha University Hospital, IRIMS, Inha University School of Medicine, Incheon 22332, Republic of Korea; 2 SCM Lifescience Co., Ltd., Incheon 22332, Republic of Korea; 3 SunCreate Co., Ltd., Yangju, Gyeonggi-do 11416, Republic of Korea; 4 Convergent Research Center for Metabolism and Immunoregulation, Inha University, Incheon 22212, Republic of Korea

## Abstract

Mesenchymal stromal cells (MSCs) are known to suppress T-cell activation and proliferation. Several studies have reported that MSCs suppress CD25 expression in T cells. However, the molecular mechanism underlying MSC-mediated suppression of CD25 expression has not been fully examined. Here, we investigated the mTOR pathway, which is involved in CD25 expression in T cells. We showed that MSCs inhibited CD25 expression, which was restored in the presence of an inducible nitric oxide synthase (iNOS) inhibitor. Since CD25 mRNA expression was not inhibited, we focused on determining whether MSCs modulated components of the mTOR pathway in T cells. MSCs increased the phosphorylation of liver kinase B1 (LKB1) and AMP-activated protein kinase (AMPK) and decreased the phosphorylation of ribosomal protein S6 kinase 1 (S6K1) and eukaryotic translation initiation factor 4E-binding protein 1 (4E-BP1). In addition, the expression of 4E-BP1 increased dramatically in the presence of MSCs. An m^7^GTP pull-down assay showed increased binding of 4E-BP1 to the 5′ cap-binding eukaryotic translation initiation factor 4E (eIF4E) complex in the presence of MSCs, which resulted in inhibition of mRNA translation. Treatment with 4EGI-1, a synthetic inhibitor of mRNA translation, also reduced CD25 expression in T cells. Polysome analysis confirmed decreased CD25 mRNA in the polysome-rich fraction in the presence of MSCs. Taken together, our results showed that nitric oxide, produced by MSCs, inhibits CD25 translation through regulation of the LKB1-AMPK-mTOR pathway to suppress T cells.

The mammalian target of rapamycin complex 1 (mTORC1) is a serine/threonine kinase that functions in mRNA translation to promote cell growth and proliferation.^1, 2^ Phosphorylation of two mTORC1 downstream targets, ribosomal protein S6 kinase 1 (S6K1) and eukaryotic translation initiation factor 4E-binding protein 1 (4E-BP1), initiates mRNA translation.^3, 4^ Translation is tightly controlled by the 5′ cap-binding eukaryotic translation initiation factor (eIF) complex,^4^ and mRNA translation is blocked when 4E-BP1 binds to eIF4E. Phosphorylation of 4E-BP1 and S6K releases them from eIF4E and eIF3, respectively. Subsequently, the binding of eIF4E, eIF4G, eIF3, and other factors to mRNA initiates translation.^3^ Two upstream targets of mTORC1 are liver kinase B1 (LKB1) and AMP-activated protein kinase (AMPK), and LKB1-AMPK signaling negatively regulates T-cell effector functions through inhibition of mTORC1.^5^ In T lymphocytes, signaling through the T-cell receptor (TCR) and CD28 activates mTORC1, which increases mRNA translation, regulates cell cycle progression, and promotes interleukin-2 (IL-2) receptor expression.^6, 7^ Thus, mTORC1 is an important regulator of T-cell proliferation, differentiation, and effector function.^2, 8, 9^

IL-2 is an autocrine mediator of survival and proliferation for T cells, and it stimulates the differentiation of naive T cells into effector T cells.^10^ The IL-2 receptor consists of three polypeptide chains, IL-2R*α* (CD25), IL-2R*β* (CD122), and IL-2R*γ* (CD132). CD122 and CD132 combine to form an intermediate-affinity IL-2R that can transmit signals, but cannot stimulate proliferation in naive T cells.^11, 12, 13^ Upon TCR ligation together with CD28, naive T cells upregulate CD25 and respond to IL-2 via the high-affinity trimeric IL-2R, which promotes T-cell proliferation.^11, 14^ Several anti-CD25 monoclonal antibodies have been developed that block interaction with IL-2 and prevent T-cell activation.^15, 16^ Some of these antibodies are currently used to treat immune disorders such as multiple sclerosis (MS) and acute graft-versus-host disease (GvHD); they are also used to for immune suppression in individuals who have received kidney transplants.^16, 17, 18, 19, 20^

Mesenchymal stromal cells (MSCs), also known as multipotent mesenchymal stem cells, exist in nearly all tissues and can differentiate into a variety of cell types.^21, 22^ MSCs inhibit immune responses through their interactions with neutrophils, macrophages, natural killer cells, dendritic cells, and B and T lymphocytes.^23, 24, 25^ The most prominent therapeutic effects of MSCs are mediated by their immunomodulatory functions.^26, 27^ Therefore, MSCs are considered a therapeutic source for the treatment of immune system disorders, such as MS,^28^ GvHD,^29, 30, 31^ type 1 diabetes,^32^ rheumatoid arthritis,^33^ systemic lupus erythematosus,^34^ atopic dermatitis,^24, 35^ and acute pancreatitis.^36^ The T-cell-immunomodulatory properties of MSCs have been the subject of studies by several research groups.^29, 37, 38, 39^ However, the mechanisms of MSC-mediated immunomodulation are complex and not yet fully understood.

One interesting observation was that MSCs suppress the expression of CD25 in T cells of both humans and mice.^40, 41, 42, 43^ However, the molecular mechanism underlying MSC-mediated suppression of CD25 is not yet been extensively examined. Since the mTOR pathway is involved in the regulation of CD25 expression,^6, 7^ we investigated whether MSCs suppress CD25 expression by regulating the downstream and/or upstream pathway components of mTORC1 signaling.

## Results

### MSCs inhibit CD25 expression

**Figure 1:**
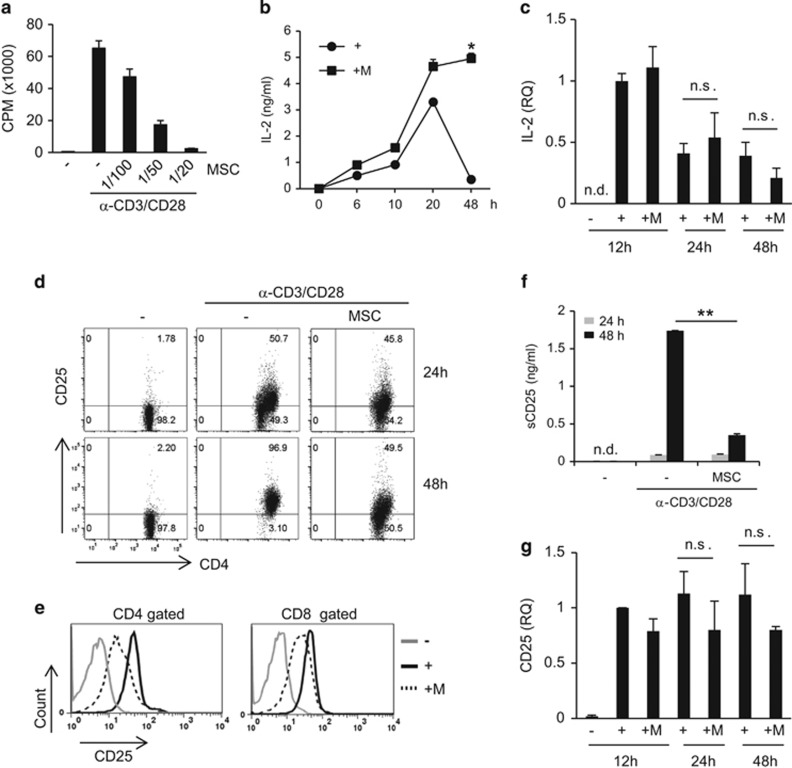
Inhibition of T-cell CD25 expression by MSCs. Lymphocytes from the spleen and lymph nodes were stimulated with anti-CD3 and anti-CD28 antibodies in the presence or absence of MSCs. (**a**) Lymphocytes (2 × 10^5^) were cultured for 3 days. During the final 16 h of culture, 1 *μ*Ci ^3^H-thymidine was added, and T-cell proliferation was determined by thymidine incorporation. The number of MSCs used was 1/100, 1/50, and 1/20 that of lymphocytes. (**b**) Lymphocytes (1 × 10^6^) were stimulated with anti-CD3 and anti-CD28 antibodies in the presence or absence of MSCs. IL-2 expression in the cell culture media was measured by ELISA. The number of MSCs used was 1/20 that of lymphocytes. (**c**) IL-2 mRNA expression was measured at different time points by qRT-PCR. Targets were normalized to 18S ribosome levels. (**d**) CD25 protein expression was measured by flow cytometry. (**e**) Intra- and extracellular CD25 expression was measured at 48 h by flow cytometry. (**f**) sCD25 protein expression in cell culture media was measured by ELISA. (**g**) CD25 mRNA expression was measured by qRT-PCR. Targets were normalized to 18S ribosome levels. Similar results were obtained in three independent experiments. −: unstimulated, +: anti-CD3 and anti-CD28 antibody-stimulated, M: MSCs. *P<0.05, **P<0.01, n.s., not significant, n.d., not detected

In agreement with previous studies, we observed that MSCs inhibited T-cell proliferation and cell division and increased apoptosis (Figure 1a and Supplementary Figure S1). Expression of inflammatory cytokines such as IFN-*γ* and IL-17A was also inhibited by MSCs (Supplementary Figure S1a). When lymphocytes were cultured with anti-CD3 and anti-CD28 antibodies *in vitro*, IL-2 expression peaked at 20 h after TCR stimulation. After 48 h of stimulation, IL-2 expression in the culture medium was significantly decreased. However, in the presence of MSCs, sustained IL-2 expression was detected at 48 h (Figure 1b). To investigate whether MSCs could induce IL-2 gene expression, we measured IL-2 mRNA levels. IL-2 mRNA expression increased 12 h after TCR stimulation and decreased subsequently; however, mRNA expression was not altered in cells cultured with MSCs (Figure 1c). Next, we measured cell surface IL-2 receptor expression. CD25 surface expression on T cells increased during stimulation, with 50% of CD4^+^ T cells expressing CD25 at 24 h, and more than 95% of cells expressing CD25 at 48 h. However, in the presence of MSCs, CD25 expression at 24 h was comparable to that in TCR-stimulated control cells, and it did not increase further (Figure 1d). We observed similar intracellular CD25 levels in CD4^+^ and CD8^+^ T cells (Figure 1e). Decreased CD25 protein expression in the presence of MSCs was confirmed by western blotting (Supplementary Figure S2a), which has been reported by several research groups.^40, 41, 42, 43^ It was reported that CD25 shedding increased through the activity of matrix metalloproteinases (MMPs) secreted by MSCs,^43^ after detecting increased soluble CD25 (sCD25) levels in culture medium in the presence of MSCs. In contrast to this finding, we did not observe increased sCD25 in the culture medium in the presence of MSCs. Instead, we observed decreased sCD25 expression (Figure 1f). This suggested that CD25 expression is reduced by MSCs through another mechanism. We measured IL-2 receptor mRNA levels and found that CD25 and CD132 mRNA expression did not differ in the presence of MSCs compared to that in the TCR-stimulated group, whereas CD122 mRNA decreased slightly with MSCs at 24 h (Figure 1g and Supplementary Figure S2b). CD122 protein expression was decreased in the presence of MSCs after 48 h, whereas CD132 protein expression levels were slightly increased (Supplementary Figure S2c). These results suggested that the decrease in CD25 and CD122 protein expression in the presence of MSCs is the cause of sustained IL-2 expression in the culture media at 48 h. To investigate whether decreased CD25 expression can affect T-cell proliferation, T cells were co-cultured with MSCs. After 48 h of culture, T cells were isolated and re-stimulated in the presence of exogenous IL-2. MSC-treated T cells were significantly less proliferative in the presence of IL-2 compared to T cells that were cultured alone (Supplementary Figure S3a). These results suggested that MSC-treated T cells are less responsive to IL-2 than T cells cultured alone, and this might be caused by decreased IL-2 receptor expression.

### Nitric oxide inhibits CD25 expression

**Figure 2:**
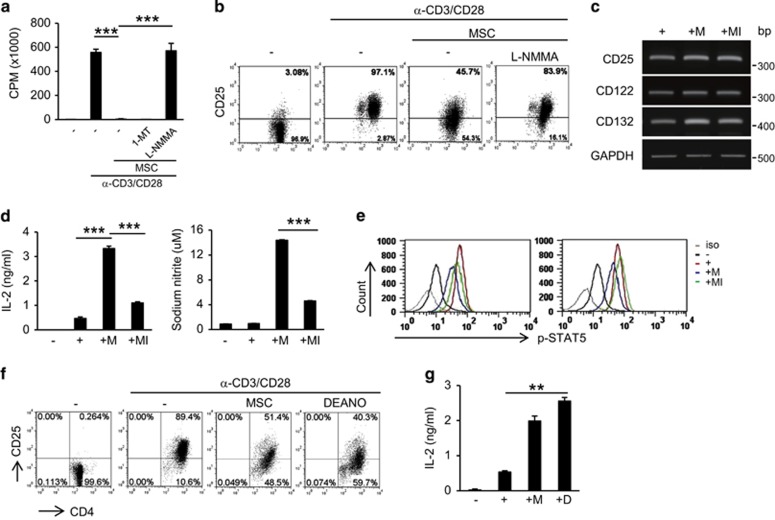
Inhibition of CD25 expression by NO. Lymphocytes were stimulated with anti-CD3 and anti-CD28 antibodies in the presence or absence of MSCs. (**a**) Cells were cultured for 3 days. During the final 16 h of culture, 1 *μ*Ci ^3^H-thymidine was added. T-cell proliferation was determined by thymidine incorporation. 1-MT: IDO inhibitor, L-NMMA: iNOS inhibitor. (**b**) Lymphocytes (1 × 10^6^) were stimulated with anti-CD3 and anti-CD28 antibodies in the presence or absence of MSCs. CD25 protein expression was measured at 48 h by flow cytometry. (**c**) CD25 mRNA expression was measured at 48 h by RT-PCR. Target levels were normalized to GAPDH levels. (**d**) IL-2 and NO levels in cell culture media were measured at 48 h by ELISA. (**e**) Intracellular phospho-STAT5 was detected at 48 h by flow cytometry. (**f**) Cells were cultured with either MSCs or the NO donor, DEANO. CD25 protein expression was measured at 48 h by flow cytometry. (**g**) IL-2 was detected at 48 h by ELISA. Similar results were obtained in two (**e**–**g**) or three (**a**–**d**) independent experiments. iso: isotype control;−: unstimulated, +: anti-CD3 and anti-CD28 antibody-stimulated; M: MSCs, MI: MSCs + L-NMMA, D: DEANO, ***P<0.001, **P<0.01 compared to the controls

**Figure 3:**
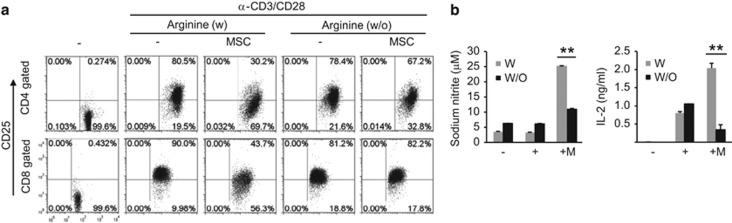
Arginine is a source of NO. Lymphocytes were stimulated with anti-CD3 and anti-CD28 antibodies for 48 h in the presence or absence of MSCs. Arginine-free RPMI 1640 media were used. (**a**) CD25 expression was detected in CD4^+^ and CD8^+^ T cells by flow cytometry. (**b**) IL-2 and NO were measured by ELISA in the cell culture media. Similar results were obtained in two independent experiments. W, with arginine, W/O, without arginine, −: unstimulated, +: anti-CD3 and anti-CD28 antibody-stimulated; M, MSCs; **P<0.01 compared to the controls

As mouse MSCs inhibit T-cell activation through nitric oxide (NO) production,^44^ we blocked NO production using L-N^G^-monomethyl arginine citrate (L-NMMA), an inducible nitric oxide synthase (iNOS) inhibitor, to investigate whether this molecule is involved in the inhibition of CD25 expression. In agreement with previously published results, we were able to rescue T-cell proliferation in the presence of L-NMMA, but not 1-methyl-D-tryptophan (1-MT), an indoleamine 2,3-dioxygenase (IDO) inhibitor (Figure 2a). CD25 expression was also restored by L-NMMA, which did not affect IL-2 receptor mRNA expression (Figures 2b and c). Increased IL-2 expression and NO production, induced by MSCs, were blocked in the presence of L-NMMA (Figure 2d). Phosphorylation of STAT5, a downstream signaling target of IL-2R, was also inhibited in the presence of MSCs and was partially or fully rescued by L-NMMA treatment in CD4^+^ and CD8^+^ T cells, respectively (Figure 2e). To test whether NO directly affects CD25 protein expression, we cultured lymphocytes with the NO donor, diethylamine NO (DEANO). This compound inhibited CD25 expression, which was associated with increased IL-2 expression, and this was similar to the effect observed in T cells in the presence of MSCs (Figures 2f and g). DEANO also inhibited T-cell division (Supplementary Figure S3b). One typical substrate of iNOS is the amino acid L-arginine; CD25 expression was not inhibited by MSCs in L-arginine-deficient medium (Figure 3a). In L-arginine-deficient medium, less NO was produced, and MSC-mediated IL-2 induction was not observed (Figure 3b). These results suggested that NO is involved in MSC-mediated inhibition of CD25 expression.

### The IFN-*γ* receptor and iNOS in MSCs are involved in the inhibition of CD25 expression in T cells

**Figure 4:**
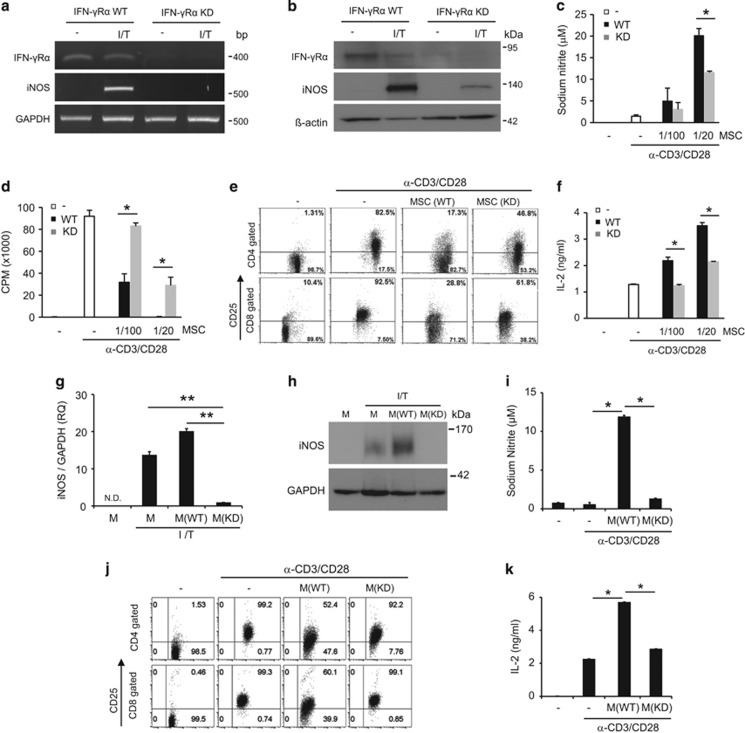
Effect of IFN-*γ*R*α* and iNOS knockdown MSCs on CD25 expression. To knock down IFN-*γ*R*α*, MSCs were infected with shRNA-harboring lentiviral particles (**a**–**f**). IFN-*γ*R*α* KD MSCs were stimulated with IFN-*γ* (20 ng/ml) and TNF-*α* (10 ng/ml). IFN-*γ*R*α* and iNOS expression was measured after 24 h by (**a**) RT-PCR or (**b**) western blotting. IFN-*γ*R*α* KD MSCs were cultured with lymphocytes for 48 h. (**c**) NO and (**d**) T-cell proliferation were measured by ELISA and thymidine incorporation, respectively. (**e**) CD25 cell surface expression was measured at 48 h by flow cytometry. (**f**) IL-2 secretion into cell culture media was measured at 48 h by ELISA. To knock down iNOS, MSCs were transfected with siRNA (**g**–**k**). iNOS KD MSCs were stimulated with IFN-*γ* (20 ng/ml) and TNF-*α* (10 ng/ml). iNOS mRNA and protein were measured after 24 h by (**g**) qRT-PCR or (**h**) western blotting. iNOS KD MSCs were cultured with lymphocytes for 48 h. (**i**) NO and (**j**) CD25 cell surface expression were measured by ELISA and flow cytometry, respectively. (**k**) IL-2 level in culture media was measured at 48 h by ELISA. Similar results were obtained in two independent experiments. WT, wild type; KD, knockdown; I, IFN-*γ*, T; TNF-*α*; M, MSCs, *P<0.05, **P<0.01 compared to the controls

Inflammatory cytokines such as IFN-*γ*, TNF-*α*, and IL-1 are required for the immunomodulatory functions of MSCs.^44^ Here, we investigated whether the IFN-*γ* receptor (IFN-*γ*R) in MSCs is involved in CD25 inhibition. Using lentiviral infection, we knocked down IFN-*γ*R*α* gene expression in MSCs. Inhibition of IFN-*γ*R*α* resulted in reduced iNOS expression in the presence of IFN-*γ* and TNF-*α* (Figures 4a and b). IFN-*γ*R*α* knockdown (IFN-*γ*R*α*KD) MSCs produced less NO than wild-type MSCs (Figure 4c). Inhibition of T-cell proliferation and CD25 expression were diminished in IFN-*γ*R*α*KD MSCs when compared to these parameters in wild-type MSCs (Figures 4d and e). Increases in IL-2 expression were also suppressed in IFN-*γ*R*α*KD MSCs compared to that in wild-type MSCs (Figure 4f). To confirm whether NO is directly involved in the CD25 expression, we knocked down iNOS gene expression using small interfering RNA (siRNA) in MSCs. iNOS mRNA and protein expression were not induced by IFN-*γ* and TNF-*α* treatment (Figures 4g and h). Under co-culture system with lymphocytes and MSCs, NO was not induced and CD25 expression was not inhibited in the presence of iNOS KD MSCs (Figures 4i and j). Increases in IL-2 expression were also suppressed in iNOS KD MSCs comparable to that in stimulated lymphocytes alone (Figure 4k). These results suggested that IFN-*γ*R*α*-mediated NO is involved in CD25 expression.

### MSCs inhibit mRNA translation signaling

**Figure 5:**
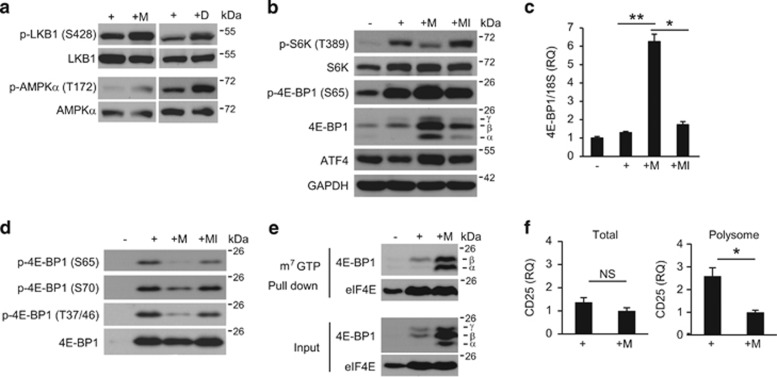
MSCs inhibit mTOR signaling. (**a**) T cells were cultured with MSCs for 24 h. T cells were stimulated for 12 h, after which DEANO was added and the cells were cultured for 2 h. Phosphorylation of LKB1 and AMPKα was detected by western blotting. (**b**) T cells were cultured with MSCs for 24 h in the presence or absence of L-NMMA. Phosphorylation of S6K and 4E-BP1 was measured by western blotting. (**c**) 4E-BP1 mRNA was measured by qRT-PCR. Target levels were normalized to 18S ribosome levels. (**d**) Phosphorylation of 4E-BP1 was measured by western blotting. (**e**) To measure 4E-BP1 binding to eIF4E, an m^7^GTP pull-down assay was performed. eIF4E-binding to 4E-BP1 was detected by western blotting. (**f**) T cells were cultured with MSCs for 24 h. Polysome-rich RNA was separated from total RNA using a 15–55% sucrose gradient system. CD25 mRNA was detected by qRT-PCR. Target levels were normalized to 18S ribosome levels. Similar results were obtained in two (**c** and **e**) and three (**a**, **b**, **d** and **f**) independent experiments. −: unstimulated, +: anti-CD3 and anti-CD28 antibody-stimulated, M: MSCs, MI: MSCs + L-NMMA, D: DEANO, NS: not significant, *P<0.05, **P<0.01 compared to the controls

Although CD25 protein was decreased in the presence of MSCs, CD25 mRNA expression was not significantly diminished (Figures 1d and g). This suggested that CD25 protein synthesis might be inhibited during early TCR signaling in the presence of MSCs. Therefore, we investigated whether MSCs can affect mTORC1 signaling, which is involved in protein synthesis. We investigated whether upstream and downstream components of mTORC1 were affected by MSCs. Specifically, we measured the phosphorylation of LKB1 and AMPK, which negatively regulate mRNA translation.^45, 46, 47^ LKB1 and AMPK*α* phosphorylation increased when T cells were cultured with MSCs or DEANO upon TCR stimulation (Figure 5a). Next, we measured the phosphorylation of 4E-BP1 and S6K. 4E-BP1 can be detected as three bands, *α*, *β*, and *γ*, depending on its phosphorylation status. The *α* and *β* forms bind to eIF4E and inhibit mRNA translation, whereas the *γ* form is released from eIF4E, which results in the initiation of mRNA translation.^3^ TCR stimulation increased the phosphorylation of 4E-BP1 and S6K (Figure 5b). In the presence of MSCs, S6K phosphorylation was inhibited and 4E-BP1 expression was strongly induced (Figure 5b). In particular, the *α* and *β* forms of 4E-BP1 were significantly upregulated in the presence of MSCs. 4E-BP1 mRNA expression was also increased in the presence of MSCs (Figure 5c), which was likely due to an increase in activating transcription factor 4 (ATF4) expression, a transcription factor that regulates 4E-BP1 (Figures 5b and c).^48^ Phosphorylation of 4E-BP1 was increased in the presence of MSCs (Figure 5b). However, when we normalized 4E-BP1 protein expression, we observed decreased phosphorylation of 4E-BP1 in the presence of MSCs (Figure 5d). The reduced phosphorylation of S6K and 4E-BP1 and the increased 4E-BP1 expression in the presence of MSCs were restored by L-NMMA (Figures 5b–d). To investigate whether binding of 4E-BP1 to eIF4E is increased in the presence of MSCs, an m^7^GTP pull-down assay was performed. As shown in Figure 5e, we observed that 4E-BP1 expression was strongly induced, and increased quantities of the *α* and *β* forms were pulled down with m^7^GTP in T cells cultured in the presence of MSCs. The expression levels of eIF4E in activated T cells in the presence or absence of MSCs were similar. To confirm MSC-mediated inhibition of mRNA translation, we isolated polysome-rich mRNA using a sucrose gradient system. We found that, although the total amount of CD25 mRNA was not affected by the presence of MSCs, the amount of CD25 mRNA in the polysome-rich fraction was significantly decreased in the presence of MSCs (Figure 5f). These results suggested that MSCs affect the LKB-AMPK-mTOR pathways.

### Blocking mRNA translation inhibits CD25 expression

**Figure 6:**
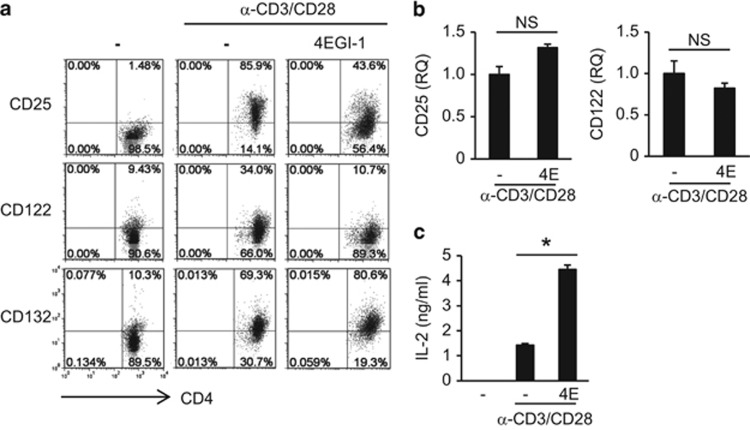
Inhibition of CD25 and CD122 protein expression by mRNA translation inhibitor 4EGI-1. Lymphocytes were stimulated in the presence of 4EGI-1. (**a**) CD122 and CD132 cell surface expression was measured at 40 h by flow cytometry. (**b**) Expression of CD25 and CD122 mRNA was measured at 20 h by qRT-PCR. Target levels were normalized to GAPDH levels. (**c**) IL-2 expression was measured at 40 h by ELISA. Similar results were obtained in two independent experiments. 4E: 4EGI-1, *P<0.05 compared to the controls

Next, we assessed whether blocking translational initiation could inhibit CD25 expression. We used 4EGI-1, a synthetic molecule that interferes with the binding of eIF4E to eIF4G after the release of 4E-BP1 from eIF4E, which ultimately inhibits mRNA translation.^4^ In the presence of 4EGI-1, we observed a decrease in CD25 and CD122 protein expression, whereas the expression of CD25 and CD122 mRNA was not affected (Figures 6a and b). IL-2 expression was significantly increased in the presence of 4EGI-1 (Figure 6c). In addition, CD132 protein expression was slightly increased following the addition of 4EGI-1, and similar results were obtained in CD8 T cells (Figure 6a and Supplementary Figure S4). These results are similar to the effects of MSCs on the IL-2 receptor complex, and they suggested that blocking mRNA translation inhibits CD25 and CD122 protein expression.

## Discussion

Inhibition of CD25 expression can explain many of the effects of MSCs on T cells. Results of previous studies, such as decreased cell proliferation, cell division, differentiation, cytokine production, and glucose metabolism,^37, 49, 50, 51, 52, 53^ as well as increased apoptosis in the presence of MSCs were probably due to a loss of the IL-2 response via the inhibition of CD25. Here, we demonstrated for the first time that MSCs suppress CD25 mRNA translation by regulating the LKB1-AMPK-mTOR pathway to potentiate T-cell suppression.

LKB1 is a serine/threonine kinase that functions as a regulator of T-cell development, activation, and metabolism.^5^ Loss of LKB1 in T cells leads to decreased AMPK phosphorylation and increased mTORC1 activation, resulting in increased T-cell activation and inflammatory cytokine production.^5^ In the presence of MSCs, we observed increased phosphorylation of LKB1 and AMPK*α* and decreased phosphorylation of S6K1 and 4E-BP1, which were restored by an iNOS inhibitor. NO causes endoplasmic reticulum (ER) stress and induces the phosphorylation of AMPK*α* via inositol-requiring enzyme 1 (IRE1).^46^ We tested whether an IRE1 inhibitor (STF-083010)^54^ could rescue the inhibition of CD25 expression in the presence of MSCs. However, the inhibitor did not rescue this effect (data not shown). As ER stress induced XBP1 mRNA splicing, we analyzed XBP1 mRNA splicing by RT-PCR; however, we could not detect the spliced form of XBP1 mRNA (data not shown). These results suggested that MSCs do not cause ER stress and that IRE1 is not involved in MSC-mediated AMPK*α* phosphorylation in T cells. As LKB1 and AMPK participate in an energy-sensing cascade,^47^ we investigated whether MSCs can affect ATP concentration. The ATP concentration in lymphocytes decreased in the presence of MSCs (data not shown). Thus, the decrease in ATP concentration induced by MSCs probably activates LKB1 and AMPK*α*, resulting in the inhibition of the mTOR pathway. In addition, 4E-BP1 protein expression was significantly increased in the presence of MSCs, which likely increased its binding to eIF4E, thus inhibiting mRNA translation. It is probably that increased 4E-BP1 expression was due to an increase in ATF4 expression. Further work will be required to elucidate the exact molecular mechanisms through which MSCs affect ATF4 and 4E-BP1 expression.

Polysomes are complex clusters of ribosomes that have a role in mRNA translation.^55^ Inhibition of CD25 mRNA translation was confirmed by polysome analysis. Whereas the total amount of CD25 mRNA was unchanged with or without MSC co-culture, the amount of CD25 mRNA in the polysome-rich fraction decreased in the presence of MSCs. We obtained similar results with the addition of 4EGI-1. Following 4EGI-1 treatment, the expression of CD25 and CD122 proteins decreased, whereas their mRNA expression was unchanged. Increased IL-2 expression was also detected in the culture medium, probably due to decreased CD25 and CD122 expression. Intriguingly, the expression of CD132 slightly increased in the presence of MSCs or 4EGI-1. CD132 expression is probably regulated differently by MSCs. Thus, MSC-mediated LKB and AMPK activation and decreased S6K1 and 4E-BP1 phosphorylation might result in decreased T-cell proliferation and inflammatory cytokine production through the inhibition of CD25 mRNA translation.

In the presence of IFN-*γ*R*α*KD MSCs, MSC-mediated CD25 inhibition was not completely rescued. It is likely that the partial knockdown of IFN-*γ*R*α* resulted in decreased NO, which could still inhibit CD25. When we knocked down iNOS genes, we could completely rescue the MSC-mediated CD25 inhibition. Using an iNOS inhibitor, and an NO donor, we confirmed that NO is involved in the inhibition of the LKB1-AMPK-mTOR pathway and CD25 expression. It has been reported that IDO is a major immunomodulatory factor for human MSCs.^56^ IDO is an enzyme that catalyzes the degradation of tryptophan. Because the inhibition of CD25 expression has also been observed in human T cells in the presence of human MSCs,^42^ it is possible that the depletion of tryptophan by MSCs inhibits the mTOR pathway,^53^ which in turn inhibits CD25 expression in lymphocytes. Determining whether the mechanisms in murine and human MSCs are similar will be investigated in future research.

Some studies have reported the inhibition of CD25 expression by MSCs,^40, 41, 42, 43^ and two studies suggested that MMPs are responsible for the cleavage of CD25. Blocking the activity of MMPs was shown to completely or partially abolish the suppression of T-cell proliferation induced by MSCs in addition to restoring CD25 expression and responsiveness to IL-2.^40^ One study showed increased sCD25 and decreased IL-2 protein concentrations in the conditioned media from cultured MSCs.^43^ In our culture system, sustained IL-2 expression in the culture media and decreased CD25 expression in lymphocytes were observed in the presence of MSCs. However, we could not detect an increase in sCD25 expression in the presence of MSCs. CD25 inhibition was restored by an iNOS inhibitor. The differences between these studies might be due to the use of different MSC lines. In previous studies, MSCs from Balb/c mice were used, whereas in the present study, MSCs isolated from C3H/HeN mice were used. We previously demonstrated that different MSC lines show variations in the inhibition of T-cell proliferation and in GvHD treatment efficacy,^31, 52^ suggesting that the immunomodulatory properties of individual MSC lines might vary. It is generally accepted that different MSC lines have different therapeutic efficacies, and that their use can result in different outcomes.

In conclusion, we demonstrated that inhibition of CD25 by MSCs is mediated by the inhibition of mRNA translation. Our findings might help to explain the therapeutic efficacy of MSCs *in vivo*; in addition, CD25 expression levels could be used as a potent marker of MSC-mediated immunosuppression.

## Materials and methods

### Mice

Balb/c and C57BL/6 mice were purchased from Orient Co. (Seongnam, Korea). All mice were maintained in a specific pathogen-free barrier facility at Inha University. All animal studies were approved by our Institutional Animal Care and Use Committee.

### Reagents

Carboxyfluorescein succinimidyl ester for T-cell division analysis and 1-MT, an IDO inhibitor, were purchased from Sigma-Aldrich (St. Louis, MO, USA). DEANO, a NO donor, was purchased from Enzo Life Science (East Farmingdale, NY, USA). L-NMMA, a NO inhibitor, was purchased from EMD Millipore (Billerica, ME, USA). 4EGI-1 was purchased from Merck Millipore (Billerica, ME, USA). Lymphocytes were cultured with 1 mM L-NMMA, 1 nM 1-MT, 1–2 mM DEANO, or 20 *μ*M 4EGI-1. CD4-FITC (RM-4-5), CD8-PE (53-6.7), CD25-APC (3C7), CD122-PE (TM-1), CD132-PE (4G3), Annexin-V-FITC, and propidium iodide were purchased from BD Biosciences (San Jose, CA, USA). RNasin was purchased from Promega (Madison, WI, USA).

### MSC culture

Mouse clonal MSCs were isolated from the bone marrow of C3H/HeN mice according to the SCM protocol^23, 24^ and were maintained in Dulbecco's modified Eagle medium-low glucose containing 10% fetal bovine serum (Gibco, Grand Island, NY, USA) and 1 × antibiotic–antimycotic solution (Gibco) at 37 °C in 5% CO_2_. Cells from passages 12 to 20 were used in this study. IFN-*γ*R*α* knockdown was induced using shRNA lentiviral particles (SC-35636-v; Santa Cruz Biotechnology, Santa Cruz, CA, USA). Twenty-four hours before viral infection, 1 × 10^4^ MSCs/well were cultured in 12-well dishes. Lentiviral particles harboring shRNA were transduced according to the shRNA Gene Silencing Protocol (Santa Cruz Biotechnology). IFN-*γ*R*α* knockdown MSCs were selected after treatment with puromycin dihydrochloride (SC-108071; Santa Cruz Biotechnology). MSCs were characterized by flow cytometry according to cell surface antigens and their differentiation potential into adipocytes, chondrocytes, and osteocytes (Supplementary Figure S5). Antibodies against CD44 (IM7), CD34 (RAM34), CD45 (30F11), CD73 (TY/11.8), CD105 (FAB1320F), CD117 (2B8), MHC II (2G9), and Sca-1 (D7) were purchased from BD Biosciences. MSCs were routinely examined for mycoplasma with a mycoplasma detection kit (e-Myco; iNtRON, Sungnam, Korea). All MSCs used were mycoplasma-free lines.

### Knockdown MSCs

To knock down IFN-*γ*R*α*, shRNA lentivial particles were purchased from Santa Cruz Biotechnology and transfected according to the manufacturer's protocol. IFN-*γ*R*α* KD MSCs were maintained using puromycin. To knock down iNOS, commercial iNOS siRNA (#SN-1001) and control siRNA (#SN-1002) were purchased from Bioneer (Dejeon, Korea). In all, 2.5 × 10^4^ MSCs/well were seeded in six-well plate 1 day before transfection. Transfection has been done using OptiMEM (Gibco) and Lipofectamine RNAiMAX reagent according to the manufacturer's protocol (Invitrogen, Carlsbad, CA, USA). After 48 h, iNOS siRNA MSCs were co-cultured with lymphocytes under anti-CD3 and anti-CD28 antibodies.

### T-cell proliferation and cytokine assays

Spleen and lymph nodes were removed from Balb/c mice and placed into a cell strainer. Using the plunger end of a 1 ml syringe, the spleen and lymph nodes were mashed through the cell strainer into a 50-ml falcon tube. The cell strainer was rinsed three times with 10 ml RPMI 1640 culture medium. Cells were centrifuged and red blood cells were removed using RBC lysis buffer (Sigma-Aldrich). To obtain T cells, B cells were depleted using anti-B220 antibodies and the MACS system (Miltenyi Biotec; Bergisch Gladbach, Germany). To investigate the effect of MSCs on T-cell proliferation, 2 × 10^5^ lymphocytes were stimulated with anti-CD3 (145-211) and anti-CD28 (37.51) antibodies (1 *μ*g/ml; BD Bioscience), along with 1 × 10^4^ MSCs (or as indicated otherwise), in a 96-well plate for 3 days. During the final 16 h of culture, 1 *μ*Ci/well ^3^H-thymidine was added, and T-cell proliferation was determined by thymidine incorporation. To detect cytokines, 1 × 10^6^ lymphocytes were stimulated with anti-CD3 and anti-CD28 antibodies (1 *μ*g/ml, each), along with 5 × 10^4^ MSCs, in a 24-well plate. The number of MSCs used was 1/20 that of lymphocytes. The numbers of MSCs used are indicated in the figure legends. After 24 and 48 h of stimulation, IFN-*γ*, IL-17A, IL-2, and sCD25 levels were measured by ELISA according to the manufacturer's protocol (BD Biosciences). To test the involvement of L-arginine, we used SILAC RPMI 1640 medium (Gibco; Thermo Fisher Scientific, Waltham, MA, USA).

### Flow cytometry

Lymphocytes (1 × 10^6^) were transferred to a V-bottom 96-well plate (Corning, NY, USA) and washed once with FACS buffer (PBS with 3% BSA). The cells were incubated with FC blockade (mAb; BD Biosciences) at 4 °C for 15 min to block Fc receptors. Cell surfaces were stained with the appropriate combinations of mAbs diluted in FACS buffer at 4 °C for 15 min, and then washed twice with FACS buffer. Intracellular proteins were stained using the cell fixation/permeabilization kit (BD Biosciences). Cells were analyzed by flow cytometry (BD FACSCalibur and BD FACS Verse) using the Flowjo software (BD Biosciences).

### NO assay

The NO concentration in culture supernatants was measured using the Griess reagent. Briefly, 50 *μ*l of Griess reagent (Sigma-Aldrich) was added to 50 *μ*l of culture medium to make a NO detection solution. Then, a nitrite standard or culture supernatant was transferred to a 96-well plate and mixed with Griess reagent. The absorbance was read at 550 nm using an automated plate reader. The NO concentration was calculated using a reference NaNO_2_ standard curve.

### Semi-quantitative and quantitative RT-PCR

Total RNA was extracted from single-cell suspensions using easy-BLUE (iNtRON). RNA was reverse-transcribed and cDNA was amplified using specific primers for GAPDH (5′-CCACTGGCGTCTTCACCAC-3′ and 5′-CCTGCTTCACCACCTTCTTG-3′), CD25 (5′-TTCGTGATGTTGGGGTTTCTC-3′ and 5′-TGTCTGTTGTGGTTTGTTGCTCT-3′), CD122 (5′-GTGGACCTCCTTGACATA-3′ and 5′-GTTTCGTTGAGCTTTGACCCTCA-3′), CD132 (5′-CTGGGGGAGTCATACTGTAGAGG-3′ and 5′-AGGCTTCCGGCTTCAGAGAAT-3′), iNOS (5′-GAGATTGGAGTTCGAGACTTC-3′ and 5′-TGGCTAGTGCTTCAGACTTC-3′), and IFN-*γ*R*α* (5′-GGTGCCTGTACCGACGAATG-3′ and 5′-AACATGGTTCCCTGGCTCTC-3′). The IL-2, CD25, CD122, CD132, 4E-BP1, and 18S primers for quantitative RT-PCR (qRT-PCR) were purchased from QIAGEN (Hilden, Germany).

### Western blot analysis

B220-depleted T cells were lysed in RIPA buffer after washing twice with cold PBS. The lysates were analyzed by standard western blotting methods. Immunoreactive bands were visualized by ECL (Santa Cruz Biotechnology). Antibodies for p70S6K, 4E-BP1, phospho-p70S6K (Thr389), phospho-4E-BP1 (Thr37/46), phospho-4E-BP1 (Ser65), phospho-4E-BP1 (Thr70), LKB1, phospho-LKB1 (Ser428), AMPK*α*, phospho-AMPK*α* (Thr172), eIF4E, ATF4, and GAPDH were purchased from Cell Signaling Technology (Boston, MA, USA). Antibodies for Grb2 (C-23), *β*-actin (C4), NOS2 (N20), and CD25 (N19) were purchased from Santa Cruz Biotechnology. For the m^7^GTP pull-down assay, T cells were stimulated with anti-CD3 and anti-CD28 antibodies for 24 h. Cell lysates were incubated with m^7^GTP sepharose (Sigma-Aldrich) for 1 h and then washed with lysis buffer. The immunoprecipitated proteins were analyzed by western blotting.

### Polysome analysis

Cells were harvested after 24 h of stimulation with anti-CD3 and anti-CD28 antibodies and washed with PBS containing cycloheximide (Sigma-Aldrich). Equal numbers of cells were lysed, and cytosolic extracts were separated using a 15–55% sucrose gradient system and ultracentrifugation.^57, 58^ Protocols were modified, and 3 × 10^7^ cells were lysed with 500 *μ*l of lysis buffer (10 mM Tris-HCl pH 8.0, 140 mM NaCl, 1.5 mM MgCl_2_, 0.5% NP-40, 1 × EDTA-free protease inhibitor, 10 mM DTT, and 500 U/ml RNasin) and centrifuged at 3000 × *g* for 2 min. Then, 10 mM DTT and 100 *μ*g/ml cycloheximide were added to the supernatant, and samples were centrifuged at 10 000 × *g* for 5 min. The supernatant was loaded onto 15–55% sucrose gradients in an MLS-50 rotor (Beckman Coulter, Brea, CA, USA) and centrifuged at 35 000 r.p.m. for 60 min. The polysome-rich fraction was analyzed by real-time RT-PCR.

### Statistical analysis

A Student's *t*-test was used to compare two independent groups in which the data were normally distributed. Statistical significance is indicated in the figure legends.
