# Supplementary material for: Mesenchymal stromal cells inhibit CD25 expression via the mTOR pathway to potentiate T-cell suppression
Source: Cell Death Dis. 2017 Feb 23;8(2):e2632–. doi: 10.1038/cddis.2017.45 (PMC5386489; doi:10.1038/cddis.2017.45)
Supplement: Supplementary Information [file cddis201745x1.docx]

**Supplementary Figure legends**

**Figure 1** MSCs inhibit T cell functions. (a) Lymphocytes (1 × 10^6^) were stimulated with anti-CD3 and anti-CD28 antibodies in the presence or absence of MSCs. Cells were cultured for 2 days. IFN-γ and IL-17A levels were detected in cell culture media by ELISA. (b) Lymphocytes were stained with CFSE and stimulated with anti-CD3 and anti-CD28 antibodies. After 3 days, cells were stained with anti-CD4-PE and anti-CD8-PerCp. Cell division was analyzed by flow cytometry. MSCs (5 × 10^4^) were cultured with lymphocytes (1 × 10^6^). (c) Lymphocytes were stimulated with anti-CD3 and anti-CD28 antibodies for 2 days and cells were stained with annexin V-FITC and propidium iodide. Apoptotic cells were analyzed by flow cytometry. Similar results were obtained in three independent experiments.

**Figure 2** MSCs inhibit IL-2 receptor expression. (a) CD25 expression was analyzed by western blotting in lymphocytes stimulated with anti-CD3 and anti-CD28 antibodies in the presence of MSCs. After 48 h, cells were lysed. Grb2 was used as an internal control protein. Similar results were obtained in three independent experiments. (b) Lymphocytes (1 × 10^6^) were stimulated with anti-CD3 and anti-CD28 antibodies in the presence or absence of MSCs. CD122 and CD132 mRNA expression were measured by qRT-PCR. Targets were normalized to 18S ribosome levels. Similar results were obtained in two independent experiments. (c) CD122 and CD132 expression analyzed by flow cytometry, at different time points, in lymphocytes stimulated with anti-CD3 and anti-CD28 antibodies in the presence or absence of MSCs. Similar results were obtained in three independent experiments. -: unstimulated, +: anti-CD3 and anti-CD28 antibody stimulation, M: MSCs. n.s.: not significant, **p* < 0.05

**Figure 3** Analysis of functional IL-2 receptor. (a) T cells were stimulated with anti-CD3 and anti-CD28 antibodies in the presence of MSCs. After 48 h, T cells were separated from MSCs and cultured for another 24 h (day 3) with or without exogenous IL-2 (1 and 5 ng/mL). T cell proliferation was determined by ^3^H-thymidine incorporation. Similar results were obtained in two independent experiments. (b) CFSE-labelled lymphocytes were stimulated with anti-CD3 and anti-CD28 antibodies in the presence of DEANO (1 mM). After 48 h, cell division was analyzed by flow cytometry.

**Figure 4** Inhibition of mRNA translation suppresses CD25 and CD122 protein expression. Expression of CD25, CD122, and CD132 was analyzed in the CD8^+^ cell population by flow cytometry in lymphocytes stimulated with anti-CD3 and anti-CD28 antibodies for 48 h in the presence of 4EGI-1 (20 μM). Similar results were obtained in two independent experiments.

**Figure 5** Characterization of MSCs. (a) MSCs were differentiated into three lineages. Staining with Alizarin Red S, Safranin-O, and Oil-red-O was used to show osteogenic, chondrogenic, and adipogenic differentiation, respectively. Scale bar = 100 μM. (b) Flow cytometry analysis of stained cell surface antigens of MSCs. MSCs were CD44-, Sca-1-, CD73-, and CD105-positive and CD34-, CD117-, CD45-, and MHC class II-negative.
