# Supplementary figures and images for: Mesenchymal stromal cells inhibit CD25 expression via the mTOR pathway to potentiate T-cell suppression
Source: Cell Death Dis. 2017 Feb 23;8(2):e2632–. doi: 10.1038/cddis.2017.45 (PMC5386489; doi:10.1038/cddis.2017.45)

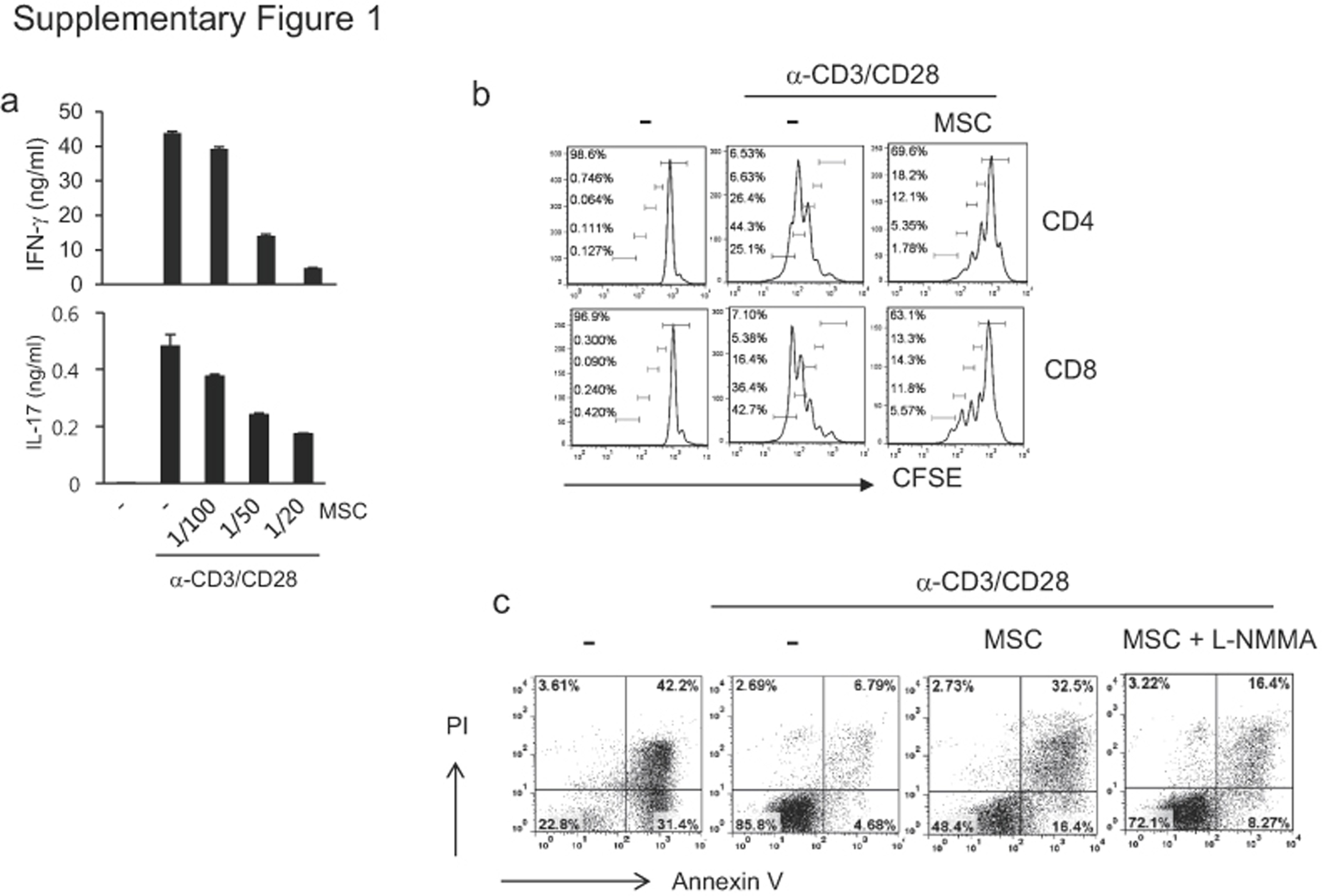

Supplement: Supplementary Figure 1 [file cddis201745x2.tif]

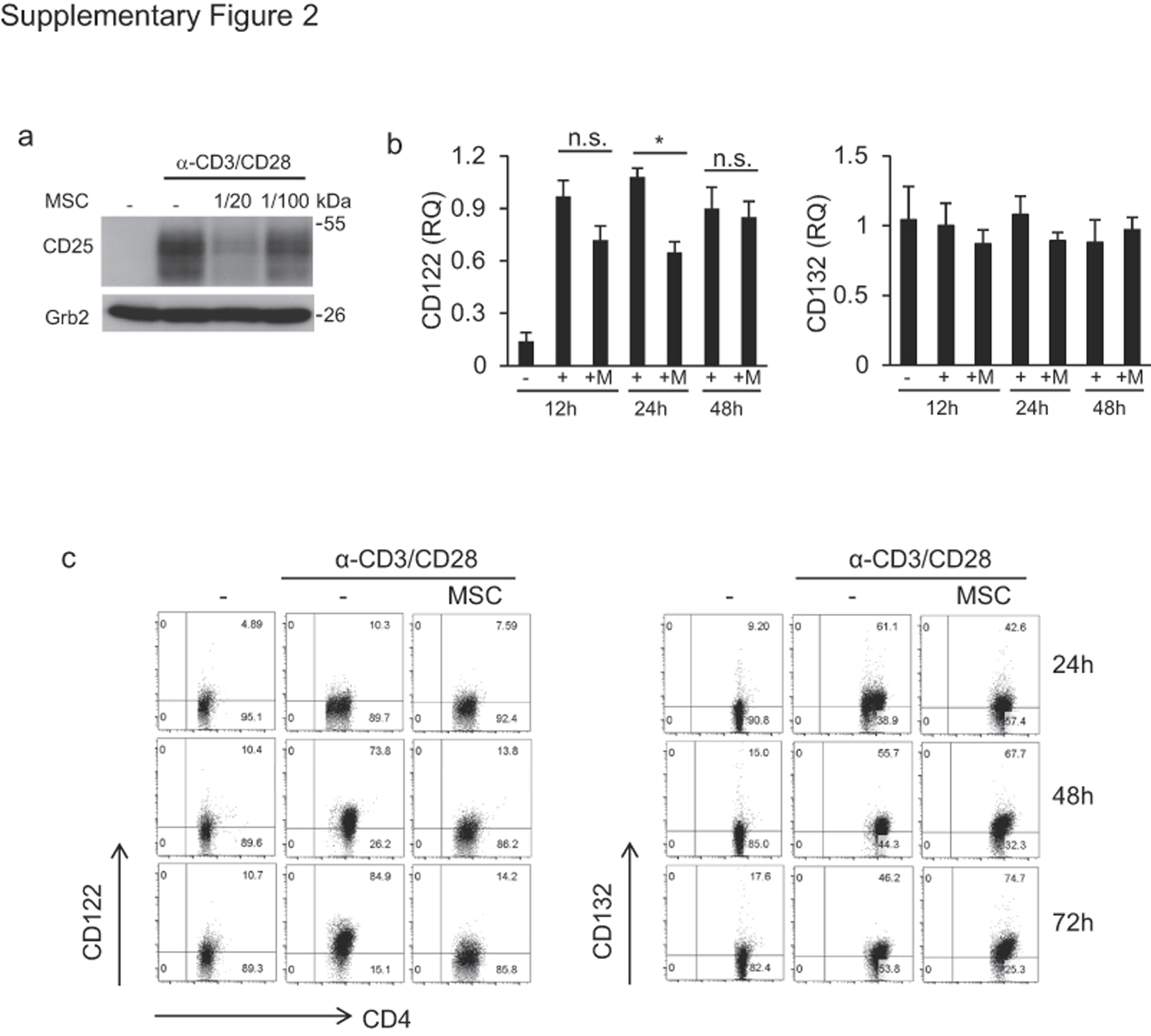

Supplement: Supplementary Figure 2 [file cddis201745x3.tif]

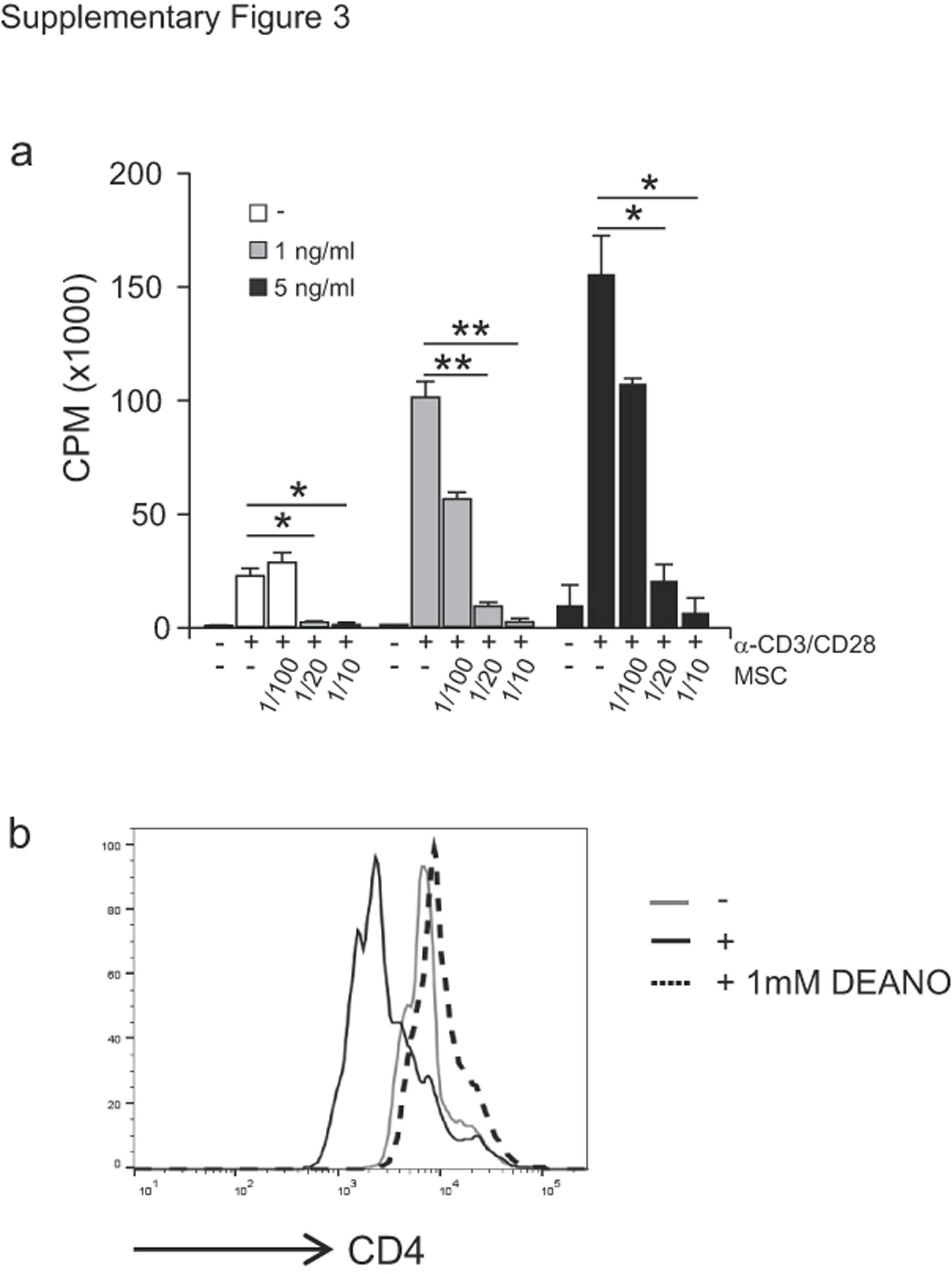

Supplement: Supplementary Figure 3 [file cddis201745x4.tif]

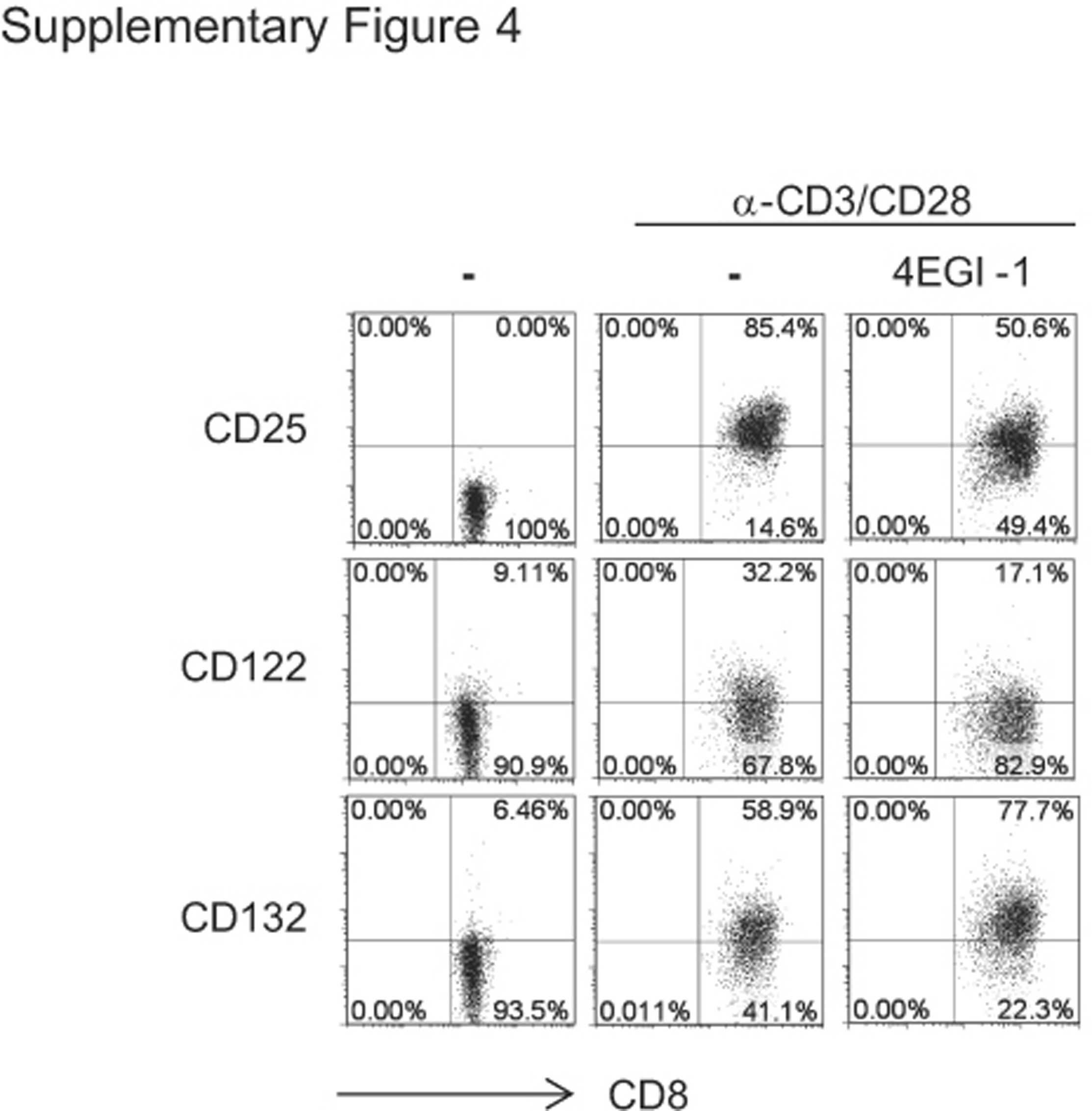

Supplement: Supplementary Figure 4 [file cddis201745x5.tif]

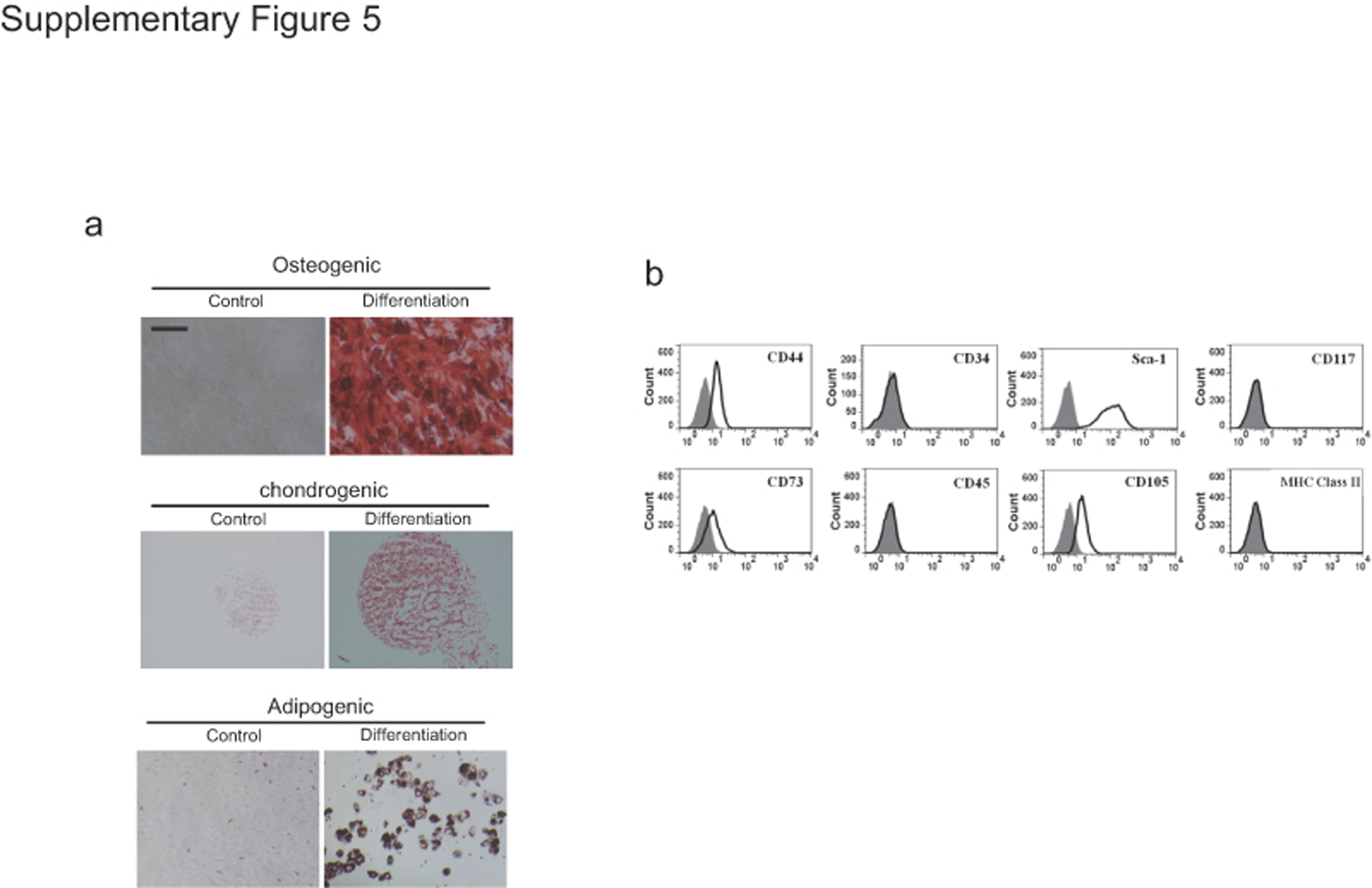

Supplement: Supplementary Figure 5 [file cddis201745x6.tif]
